# Human and Zoonotic Dermatophytoses: Epidemiological Aspects

**DOI:** 10.3389/fmicb.2021.713532

**Published:** 2021-08-06

**Authors:** Esther Segal, Daniel Elad

**Affiliations:** ^1^Sackler School of Medicine, Department of Clinical Microbiology and Immunology, Tel Aviv University, Tel Aviv, Israel; ^2^Department of Clinical Bacteriology and Mycology, Kimron Veterinary Institute, Bet Dagan, Israel

**Keywords:** dermatophytes, dermatophytoses, epidemiology, infection sources, demographic factors

## Abstract

**Introduction:**

Dermatophytes are a group of molds characterized by the ability to produce keratinases, thereby carving out for themselves specific ecological niches. Their traditional division into three genera, *Trichophyton*, *Microsporum*, and *Epidermophyton* has been expanded to nine and the species in each genus were modified. Dermatophytes are among the most prevalent causes of human and animal mycoses. Their epidemiology is influenced by various factors. These factors may be evolutive such as the predilected environment of the fungus, namely, humans (anthropophilic), animals (zoophilic), or environment (geophilic), is evolutionary and thus may require centuries to develop. Many other factors, however, result from a variety of causes, affecting the epidemiology of dermatophytoses within a shorter time frame.

**Objective:**

This review aims at summarizing the factors that have modified the epidemiology of dermatophytoses during the last decades.

**Results:**

Geographic and climatic conditions, demography such as age and gender, migration, socio-economic conditions, lifestyle, and the environment have had an impact on changes in the epidemiology of dermatophytoses, as have changes in the pattern of human interaction with animals, including pets, farm, and wild animals. A typical example of such changes is the increased prevalence of *Trichophyton tonsurans*, which spread from Latin America to the United States and subsequently becoming a frequent etiological agent of tinea capitis in Africa, Middle East, and other areas.

**Conclusion:**

The comprehension of the epidemiology of dermatophytoses has a major bearing on their prevention and treatment. Since it is undergoing continuous changes, periodic assessments of the most recent developments of this topic are required. This article aims at providing such an overview.

## Introduction

Dermatophytes is a group of molds that have carved themselves a niche by producing enzymes that dissolve keratin in skin, hair, nails, and feathers and use the resulting products as a source of nutrients (Padhye and Summerbell, 2005). The dermatophytes include, molds utilizing human keratinous tissues – the anthropophiles, causing skin, nails, and hair infections, those that utilize keratin of animal tissues – the zoophiles, and those which dwell in soils and use keratin of debris found in soil – the geophiles (Hay, 2005; Richardson and Warnock, 2012). Taxonomically, the dermatophytes belong to the order *Onycogenales*, family *Arthrodermataceae* and were until recently divided into three genera, namely, *Trichophyton*, *Microsporum*, and *Epidermophyton*. Recently, the taxonomy and nomenclature of the dermatophytes was extensively revised (de Hoog et al., 2017), based on their molecular analysis and policy of “one fungus – one name” whereby the anamorphs (asexual stages) and teleomorphs (sexual stages) must have a single designation.

Human infections can be caused by anthropophilic and zoophilic dermatophytes and occasionally, also by geophilic species (Hay, 2005). The latter, however, are contracted as a rule from the environment and not by transmission between humans or from animals to humans (Weitzman and Summerbell, 1995; Dolenc-Voljč and Gasparic, 2017); geophilic dermatophytes are not addressed in this review. An exception is *Nannizia gypsea* (formerly *Microsporum gypseum*) a fungus that causes dermatophytoses more frequently than other geophilic dermatophytes. The clinical manifestations of dermatophyte infections are generally termed “tinea” with the indication of the anatomical area involved. Thus, infections of hair on the scalp or beard are termed “tinea capitis” and “tinea barbae”, respectively, those of nails “tinea unguium” and those of glabrous skin “tinea corporis” or “tinea pedis” and “tinea manuum,” in case of foot or hand involvement, respectively (Hay, 2005).

The infections caused by dermatophytes (dermatophytoses) are among the most frequent human infections (Hay et al., 2014). The current arsenal of antifungal drugs includes preparations for topical and systemic administration (Gupta et al., 2017a; Khurana et al., 2019), the latter primarily for treatment of hair and nail infections. The latter pose a major therapeutic problem, as the treatment may require a substantial time and may be associated with adverse effects, particularly in the prone population of elderly with various co-morbidities (Thomas et al., 2010; Gupta et al., 2014).

The following review article, will focus on the epidemiology of human and zoonotic dermatophyte infections. The article will include exploration of the aspects of variation in incidence of dermatophytoses in different geographic or climatic areas, dermatophyte species distribution and relationships between gender and age to prevalence. Correlation between incidences of the various clinical entities: the skin, nail, and hair tineas and other factors, such as professional occupation, will be reviewed. In addition, aspects of the effect of migration, climate change and socio-economic conditions on epidemiology of dermatophytoses, will be explored as well. Moreover, the epidemiology of human infection with zoophilic and selected geophilic dermatophytes will be addressed.

## Human Dermatophytoses

### Incidence and Dermatophyte Species Distribution in Different Geographic/Climatic Regions

In the last decade, a number of publications from different geographic areas focusing on dermatophytes’ species epidemiology reported data from Eastern Europe (Colosi et al., 2020) and the Balkan (Otašević et al., 2019; Sakkas et al., 2020), from the Middle East (Ozkutuk et al., 2007; Segal et al., 2015; Armon et al., 2020), Africa (Coulibaly et al., 2018), South East Asia (Do et al., 2017), and South America (Silva-Rocha et al., 2017; Carrascal-Correa et al., 2020).

The data reported in the different publications shows variability and are difficult to compare, since distribution of species may also depend on the human site screened, e.g., tinea capitis or tinea corporis that may reveal different species distribution.

Thus, the reader has to keep in mind all these limiting considerations regarding the presented comparisons.

Having said all of the above, the mentioned publications reveal that both in Vietnam (Do et al., 2017) and in Africa (Coulibaly et al., 2018), two different continents, the majority of cases were the result of infection with anthropophilic dermatophytes. In Vietnam (Do et al., 2017), *Trichophyton rubrum* complex was the most common species (66.9%) followed by *Trichophyton mentagrophytes* (formerly *Trichophyton interdigale*) and *Trichophyton tonsurans* in skin infections, while in Africa (Coulibaly et al., 2018), *Trichophyton violaceum* was most common in tinea capitis. Thus, the common denominator is human–human contact as source of infection.

In a different geographic region – the Middle East (Israel), a study of the author’s group (Armon et al., 2020) in a specific demographic group: soldiers, revealed that the anthropophilic dermatophytes *T. rubrum* complex and *T. mentagrophytes* took the lead. *T. rubrum* complex was the causative agent in over 90% of nail and skin infections among soldiers.

These data from three geographical/climatic different regions: Middle East, South East Asia, and Africa, which show similar trends in dermatophyte species distribution, may indicate no specific correlation between geographic/climatic conditions and dermatophyte species distribution. Another study in Israel of the author’s group (Segal et al., 2015) on onychomycosis revealed the same trend.

A recent study on onychomycosis from North West Greece (Sakkas et al., 2020) showed that the most frequently isolated dermatophyte was *T. rubrum* complex (74.4%), followed by *T. mentagrophytes* (*T. interdigale*) (21.4%). Contrary to these findings, a study from Serbia (Otašević et al., 2019) reported that most cases were caused by a zoophilic dermatophyte – *Microsporum canis*. Thus, although both studies are basically from similar geographic areas, they differ regarding dermatophyte distribution, which may probably depend on different demographic population tested.

Regarding connection between climatic influences and prevalence of dermatophytoses, it is generally assumed that since dermatophytes grow well in humid and warm conditions, the prevalence of dermatophytoses might be higher in countries located in tropical/subtropical regions, with humid and hot climates (Havlickova et al., 2008; Coulibaly et al., 2016).

### Demographic Aspects of Dermatophytoses and Dermatophyte Species Incidence and Distribution

#### Age and Gender

Age of a screened population for a specific morbidity may be considered as a demographic parameter, which delineates the certain population group from other groups. With respect to dermatophytoses, age may indeed play a role as to prevalence of a specific dermatophytosis in a certain age group.

Thus, tinea unguium is more prevalent among older patients (Lipner and Scher, 2019), while tinea capitis is seen more frequently in children. Lipner and Scher (2019) cite a rate of nail infection of ∼6%% in the older population vs. 1.4% in children. In their extensive review on nail infections (onychomycosis), Lipner and Sher indicate advanced age as one of the risk factors for this infection. Advanced age is frequently associated with other co-morbidities such as diabetes or/and blood circulation problems (Thomas et al., 2010; Sakkas et al., 2020), which may increase the risk for dermatophyte involvement.

Although nail infections (onychomycosis) can be caused by various molds and by *Candida* species, those caused by dermatophytes are the dominant fungal group isolated from infected nails (Lipner and Scher, 2019). This was also shown by our studies surveying a large cohort in Israel (Segal et al., 2015). Here too, *T. rubrum* complex was the most prevalent dermatophyte. It is believed that in many instances, tinea pedis may be the source for the involvement of the toe – nails.

In this context, an interesting observation was made by Gnat et al. (2019b) in their recent article, suggesting involvement of specific host factors affecting the outcome of the interplay between host and pathogen in dermatophyte infection.

Another demographic parameter would be gender: female vs. male of a studied group in respect to a certain morbidity. Thus, the study of the Israeli onychomycosis cohort (Segal et al., 2015) revealed differences as to morbidity site in dermatophytoses. While toenails were infected more frequently in males than in females, the finger nails were more affected in females than in males. There were also differences noted in the fungal etiology according to gender: while in men, the great majority were infected by dermatophytes, in women, the proportion of infections with *Candida* spp. was higher than in men. Differences according to gender regarding dermatophytoses were also noted by a number of other investigators (Gupta et al., 2017b; Lipner and Scher, 2019; Sakkas et al., 2020).

#### Migration

Migration plays a major role in demographic changes. In the context of dermatophytes, it can be exemplified by *T. tonsurans* and other species, such as *Trichophyton soudanense*. *T. tonsurans* is the classical example, originating from South East Asia and Australia (Rippon, 1985, 1988), it spread to Latin America and from there with work immigrants to North America and to other parts of the globe, including the African continent.

While older studies (Rippon, 1985, 1988) from the USA indicated the rareness of this fungus reported as cause of tinea capitis, more recent studies show a high incidence. Unpublished recent data in Israel show the same trend, indicating that currently tinea capitis cases caused by *T. tonsurans* are increasing. Seebacher et al. (2008) in a detailed large review indicated that in the United States during the period between 1979 and 1995, there was a decline in frequency of *T. rubrum* complex from 53.7 to 41.3% and an increase in *T. tonsurans* from 27.9 to 44.9%.

An interesting study by Wilmington et al. (1996) described the increase in the San Francisco area during 20 years between 1974 and1994, in tinea capitis cases by *T. tonsurans*, showing a dramatic increase. While in 1970s, 41.7% of cases were caused by *T. tonsurans*, in the 1990s, the prevalence increased to 87.5%.

An interesting study on the epidemiology of *T. tonsurans* in Japan was reported by Hiruma et al. (2015). Hiruma and colleagues indicate that the first cases to be caused by this fungus in Japan appeared in the early 2000s. The number of cases increased within a short period and appeared primarily among judo-club members. The prevalence of infections by *T. tonsurans* kept increasing also in other settings, such as school age children. Moreover, infections included both symptomatic clinical cases and non-symptomatic carriers, a situation which makes evaluation of prevalence difficult. The authors recommend particular awareness in individuals in combat sports and their contacts.

In this context, it is of interest to mention the study by Gits-Muselli et al. (2017), describing a large survey on tinea capitis among immigrants’ children in the Paris area. The authors found that the three most prevalent dermatophytes were *T. soudanense*, *T. tonsurans*, and *Microsporum audouinii*. Moreover, the authors indicate that during the period of the survey (5 years), there was an increase in cases caused by *T. tonsurans*, vs. those caused by *T. soudanense* or *M. audouinii*, particularly among African immigrants vs. immigrants from the Caribbean Islands.

A recent report from the US (Grigoryan et al., 2019) also confirms that *T. tonsurans* is a major cause of pediatric tinea capitis (95% of cases). In addition, this report also indicates that two other dermatophytes: *T. violaceum* and *T. soudanense* are also significant causes of tinea capitis in children in association with migration, specifically in African immigrants.

A tinea capitis outbreak was also reported in children of African immigrants in Israel in 2016 (Mashiah et al., 2016). During the period 2010–2014, 145 children were included in the study revealing that *T. violaceum* and *M. audouinii* were the major causative agents.

#### Socio-Economic Factors

A literature search on the possible effects of socio-economic factors on dermatophytoses resulted in over a dozen publications. The search included publications as far as the 1970s until the present and represented a wide geographic range. Data were collected in Turkey (Inanir et al., 2002; Kiraz et al., 2010), India (Ranganathan et al., 1997–1998; Sarma and Borthakur, 2007; Patro et al., 2019), Cameroon (Nkondjo Minkoumou et al., 2012), Tunisia (Zahaf et al., 1979), Egypt (Farag et al., 2018), Nigeria (Ogunbiyi et al., 2005), and Israel (Leibovici et al., 2009). A number of these studies focused on infections in children: school age children in Turkey (Inanir et al., 2002), in Israel (Leibovici et al., 2009), and in Nigeria (Ogunbiyi et al., 2005). The common denominator in these reports was the effect of low socio-economic status associated with large families in crowded living conditions, poor hygiene possibilities, and association with increased prevalence of dermatophytoses. Ranganathan et al. (1997–1998) found that 35% of the infected cases were from the very low-income group (group-I), 34.2% from low-income group (group-II), 23.3% from middle-income group (group-III), and 1.8% from moderately rich group (group-IV). Unsurprisingly, the distribution of dermatophyte species involved, pointed primarily to the anthropophilic species, most frequently to the *T. rubrum* complex (Ranganathan et al., 1997–1998; Leibovici et al., 2009).

### Human Dermatophytoses and Environment

As indicated afore, classical division of dermatophytes consisted of anthropophilic, zoophilic, and geophilic dermatophytes. The latter, thriving in soil may be considered as environmental fungi. One of these, *N. gypsea*, is known as a cause of human infections (Segal and Frenkel, 2015), which indicates that environment may be considered as a source for human dermatophyte infections, as well.

Furthermore, surveys on environmental fungi, such as studies exploring the presence of fungi in sand of beaches around seas and other water bodies (Brandao et al., 2021), have shown that dermatophytes can be isolated. A reservation is due in this context, as it may be assumed that the presence of dermatophytes in these ecological sites originates from debris shed by humans frequenting these sites them (Sabino et al., 2011). In this context, it is also of interest to state that such findings are rare, and parallel studies in other areas, such as the Eastern Mediterranean area (Frenkel et al., 2020), have not corroborated these data, possibly due to differences in climatic factors, such as higher temperature and longer sunshine time in the environment in the Mediterranean area (Amichai et al., 2014).

*Nannizia gypsea* infections are more prevalent in South America and Asia than in North America or Europe with higher incidence in rural areas (Kwon-Chung and Bennett, 1992). It is the most prevalent geophilic dermatophyte causing dermatophytosis and while a large variety of animals have been reported to be carriers of the fungus, clinical human or veterinary cases are relatively rare (Ginter, 1989; Dolenc-Voljč and Gasparic, 2017). It thrives especially in soils rich in organic matter (Ginter, 1989). Thus, farmers and gardeners are populations at risk (Chmel and Buchvald, 1970). Dolenc-Voljč and Gasparic (2017) described a large survey consisting of 226 cases from Slovenia caused by *N. gypsea* including skin, hair, and nail infections. The authors indicate that contact with soil has been involved in many of the cases. An additional observation in the study is that many of the cases were in children. da Silva Souza et al. (2016) reported a study on *N. gypsea* infections in infants in which there was contact with sand. A more recent study from Korea (Lee et al., 2018) confirms the validity of these statements by affirming the continuous occurrence of infections by *M. gypseum*.

## Zoophilic Dermatophytes and Zoonoses

Dermatophytes are the most prevalent fungi isolated from animals, carriers or clinical cases (Moretti et al., 2013). The periodical changes between the prevalence of anthropophilic and zoophilic species in the etiology of human dermatophytoses were not observed in animal infections. Thus, animal dermatophytoses are caused by zoophilic or geophilic dermatophytes, with rare exceptions, mostly *T. rubrum* complex, assumed to be the result of anthropo-zoonotic transmission (Ranganathan et al., 1997–1998; Mitra et al., 1998).

As a rule, young animals are at a higher risk of infection (Cafarchia et al., 2004), possibly due to the composition of skin secretions and/or the maturity of their immune system. Some zoophilic dermatophytes show some levels of preference for a specific host group, whereas other may infect a large variety of animals.

The epidemiology of human infections with zoophilic dermatophytes depends largely on the closeness and intensity of their interaction with animals. Such contacts have different characteristics in urban, rural, or sylvatic areas. The demarcation between the three is, however, not clear-cut, as will be detailed below. Inter-human transfer of zoophilic dermatophytes, occasionally leading to outbreaks, mostly nosocomial, has been reported. Several of these outbreaks were in highly susceptible subjects, namely, neonates, infected by the attending personnel (Mossovitch et al., 1986; Rodriguez-Contreras et al., 1987; Snider et al., 1993; Drusin et al., 2000), whereas another might have been related to a contaminated fomite (electric razor) (Shah et al., 1988).

Zoophilic dermatophytes species include *M. canis*, *Nannizia nana*, *Nannizia persicolor*, *Trichophyton vanbreuseghemii*, *Trichophyton benhamiae*, *Trichophyton erinacei*, *Trichophyton quinckeanum*, *Trichophyton simii*, *Trichophyton verrucosum*, *Trichophyton equinum*, and *Lophophyton gallinae*. The zoonotic potential of these fungi is not equal, and thus the prevalence of human infections with each varies.

Zoophilic species of *T. mentagrophytes* are now divided into two species: *T. benhamiae* and *T. vanbreuseghemii* (Baert et al., 2020). This modification is significant from the historic-epidemiologic aspects since epidemiological data predating the nomenclature modification become unclear: past isolates of zoophilic *T. mentagrophytes* may be *T. benhamiae* or *T. vanbreuseghemii* and the only (ambiguous) indication as to their reclassified taxonomy may be the animal with which they were associated: *T. benhamiae* from rodents and lagomorphs and *T. vanbreuseghemii* from other animals. Thus, for example, when owners of guinea pigs were infected by “*T. mentagrophytes*” (Mazur et al., 2018), it is likely but not certain that the infection was caused by *T. benhamiae* since it is associated predominantly with rodents, primarily guinea pigs, especially when held in large groups (Berlin et al., 2020). Consequently, whenever the binomium “*T. mentagrophytes*” is used in the context of animal isolates, it refers to the zoophilic species and not the anthropophilic species, as it is currently classified.

*Trichophyton mentagrophytes* has a very wide gamut of animal hosts, including pets, farm, laboratory, and wild animals (Mariat et al., 1976; McAleer, 1980; Mitra et al., 1998). As for the two newly classified zoophilic dermatophytes, publications subsequent to the classification changes indicate that *T. benhamiae* may be isolated, in addition to rodents and lagomorphs, from other animal species such as dogs (Sieklucki et al., 2014; Scarpa et al., 2021), porcupines (Needle et al., 2019), or hedgehogs (Gregory and English, 1975). Animals may be symptomatic or asymptomatic and constitute a potential source of human infection (Gregory and English, 1975; Ansari et al., 2021). Two colony color variants, white and yellow, have been identified. It is an important source of human dermatophytosis in Germany (Bartosch et al., 2018) with the yellow variant isolated from all but a few of these cases (Berlin et al., 2020). *T. vabreuseghemii* was isolated from dogs, cats (Monod et al., 2014), and horses (Chollet et al., 2015) and has been implied in human contagions. Feral, hunting cats were found to be more frequently infected than domestic cats, which points to the possibility that *T. vanbreuseghemii* is contracted from rodents or soil (Drouot et al., 2009).

### Urban Areas

Several animal groups that act as sources of human infection with dermatophytes in the urban area are primarily stray animals and household pets, keeping of which has become one of the hallmarks of affluent societies.

The main source of human dermatophytes in urban areas in which stray animals are involved is cats being infected or, more frequently, carrying *M. canis*. The carriage of *M. canis* by cats has been assessed in many cities, resulting in up to 100% positive animals in surveys conducted in Italy (Cafarchia et al., 2004), Iran (Khosravi and Mahmoudi, 2003), or Germany (Wiegand et al., 2019), and is frequently transferred to other animals and humans. Environmental factors such as climate may have an impact on the prevalence *M. canis* infections (Cafarchia et al., 2004). The fungus was found to be highly prevalent in feral cats (McAleer, 1980) or cats held in groups such as shelters or breeding facilities (Seyedmousavi et al., 2018) due to reinfection cycles. *M. canis* is transmitted by direct contact with infected animals or indirectly by fomites or hair that, due to the cats intensive grooming habits, abound in their environment (Frymus et al., 2013). Moreover, human contagion source may be the environment as shown in an outbreak in children that contracted the infection in an open-air public swimming pool in Italy (Moretti et al., 2013).

The prevalence of the carriage of other dermatophytes by stray cats is significantly lower: Romano et al. (1997) isolated in Siena, Italy, dermatophytes from 86 (49.7%) of 173 asymptomatic stray cats. Of these, *M. canis* was isolated from 82 cats, *T. mentagrophytes* (probably *T. vanbreuseghemii*) from three cats, and *N. gypsea* from one cat. Somewhat different, but comparable, prevalence ratios were found in other surveys such as those conducted in Barcelona, Spain (Cabañes et al., 1997), and Mexico City (Guzman-Chavez et al., 2000).

In a survey of a few hundred cats and dogs from households, shelters, pet shops, or stray, conducted by Yamada et al. (2019) in Japan, *M. canis* was absent or had a very low prevalence (1.1–3%) in the surveyed areas. The fungus was, however, more prevalent (21.5%) in cats in animal raising establishments. Owners were infected by the fungus in 18.7% of the affected households. The authors note that prevalence rates vary significantly in time, possibly indicating the spread of *M. canis* from some reservoir, subsequently declining.

Interestingly, while the prevalence of *M. canis* in dogs and cats in Iran was high (Khosravi and Mahmoudi, 2003), the prevalence of human infections with the fungus was found to be relatively low, possibly due to the population, being Muslims, who do not keep pets (Naseri et al., 2013). A similar observation was made by Ng et al. (2002) in Malaysia.

### Rural Areas

Under intensive food animal raising practices (Begum and Kumar, 2020), animals are more crowded and in closer contact with humans leading to higher prevalence of dermatophytoses in both. *T. verrucosum* is the dermatophyte with the highest prevalence in ruminants (Khosravi and Mahmoudi, 2003). Humans contract infection through contact with cattle or environmental contamination with arthroconidia, which may be infective for several years, especially in conditions of high humidity (Courtellemont et al., 2017). Such contacts with animals cannot always be found (Wollina et al., 2018). Prevalence in humans may be relatively low: *T. verrucosum* was isolated from only 1.5% of 2674 patients (Courtellemont et al., 2017) in France, 1.4% of 560 patients in Iran (Naseri et al., 2013), and 2.6% of 116 surveyed farmers in east Poland (Śpiewak and Szostak, 2000) but may also reach much higher rates (16% in Ethiopia or 33% in Iran) (Moretti et al., 1998). The same authors found, while surveying the prevalence of dermatophytosis in cattle, that 19% of intensively bred beef cattle were infected with *T. verrucosum*, whereas the infection’s prevalence on traditional farms was only 8%.

Another dermatophyte associated with intensive animal raising enterprises is *N. nana*. *N. nana* causes dermatophytosis in pigs but was reported in dogs and ruminants as well (Begum and Kumar, 2020). Human infections have been reported from various geographical areas but are relatively rare (Bonifaz et al., 2019). They are usually (Roller and Westblom, 1986), but not always (Ramon-Torrell et al., 2020), a consequence of contact with infected pigs (Porras-López et al., 2020).

Horses too may be the source of human dermatophytosis, caused by *T. equinum*, albeit rarely (Veraldi et al., 2018). Transmission is by direct contact or through fomites of infected animals (Overy et al., 2015), and thus people in direct contact with horses and their environment such as riders, stable personnel, and veterinarians are at the highest risk (Huovinen et al., 1998). Moreover, human infection following contact with an asymptomatic dog has been reported (Gnat et al., 2020).

*Trichophyton quinckeanum* causes mouse favus in rodent but can be transmitted to human directly or through a variety of other animals, frequently cats (Uhrlaß et al., 2018; Wiegand et al., 2019). A massive outbreak of human dermatophytosis caused by *T. quinckeanum* was reported to have occurred in rural areas, heavily infested by rodents, in Hungary in the mid-1900s (Szathmáry, 1966). Many of these mice were showing skin lesions associated with *T. quinckeanum*, namely, mouse favus. These rodents disseminated the fungus to the environment and infected cats preying on them, leading to the human outbreak.

Another zoophilic dermatophyte that may cause rare human infections is *L. gallinae*. The elective hosts of this dermatophyte are chicken, with sporadic cases in other animals and humans (Londero et al., 1969; Yamaguchi, 2019). Among the few reported human infections, predisposing conditions such as diabetes or immunosuppressive diseases were observed in several cases (del Palacio et al., 1992; Poblete-Gutiérrez et al., 2006).

Rabbit farms may be affected by dermatophyte infection outbreaks. *T. mentagrophytes* (old classification) is the most prevalent fungus involved, with adult animals usually asymptomatic carriers, posing a significant contagion risk for the personnel (Moretti et al., 2013).

### Wild Animals

Dermatophytes may be transmitted from wild animals to humans under several conditions. Among these are hunting and truffle dogs, in contact with infected wild animals and their habitat, subsequently infecting not only their owners but other human contacts in urban or rural areas. Another source of human dermatophytosis is wild animals encroaching human dwellings due to the restriction of the formers’ living areas by urbanization and/or agricultural activities (Moretti et al., 2013).

Several surveys of keratinophilic fungi in wild animals and their environment resulted in the isolation of a large variety of such microorganisms (Alteras et al., 1966; Chabasse, 1988). Mantovani et al. (1982) found fungi belonging to the *T. mentagrophytes* complex, *M. canis*, and the *N. gypsea* complex to be the most common. *T. terrestre* and *T. mentagrophytes* were isolated from the coats of wild boars in Italy (Mancianti et al., 1997). *T. mentagrophytes* and *N. gypsea* were isolated from Florida panthers (Rotstein et al., 1999), a non-speciated *Trichophyton* species from two wild felines in Brazil (Albano et al., 2013) and *N. gypsea* from adult impalas and Grant’s gazelles in Kenya (Nweze and Eke, 2018). In two separate surveys, one of marmots in Switzerland and another of Eastern cottontail in Italy, six species of dermatophytes were isolated, including *M. canis* and *T. mentagrophytes* and *N. gypsea*, *Paraphyton cookei*, *Trichophyton ajelloi*, and *Trichophyton terrestre* (geophilic), respectively (Gallo et al., 2005a, b). *T. mentagrophytes* was isolated from free ranging red foxes in the United States (Knudtson et al., 1980) and from animals a silver fox breeding farm in Poland (Gnat et al., 2019a). Conversely, Hall et al. (2011) found no dermatophytes in a survey of White-tailed Deer in Virginia, United States.

In some cases, wild animals are raised as pets: keeping hedgehogs as pets is popular in Japan and a source of *T. erinacei* infection in humans (Takahashi et al., 2003; Rhee et al., 2009). Another source of human exposure to dermatophytes in sylvan areas is tourism: Gallo et al. (2005a) isolated from marmots in Italy, in areas frequented by tourists, *M. canis*, *T. mentagrophytes*, *P. cookei, N. gypsea*, *T. ajelloi*, and *T. terrestre*.

*N. persicolor* has been reported most frequently from European countries (Stockdale, 1953) but was isolated in India, South Africa, and North America (Padhye and Ajello, 1974; Kane et al., 1987) as well. Interestingly, *N. persicolor* infects skin and not hair, a fact that possibly limits its environmental dispersal (Wiegand et al., 2019). *N. persicolor* is a zoophilic dermatophyte carried primarily by sylvatic rodents (Mariat et al., 1976) such as voles and mice and is present in the environment populated by these animals. Consequently, hunting dogs are the most likely to be infected (Muller et al., 2011). The fungus was, however, isolated from various other animal species in other surroundings (Wiegand et al., 2019), including bats (Onsberg, 1978). Several human dermatophytoses caused by this fungus were reported (Krzyściak et al., 2015; Metzner et al., 2018). In one report, both husband and wife were infected. They had contact with animals showing skin lesions (not examined), and human to human transmission was deemed unlikely (Wiegand et al., 2019).

In addition to rodents and lagomorphs, other animal species may be carriers or infected with *T. benhamiae*, including dogs (Sieklucki et al., 2014; Scarpa et al., 2021), porcupines (Needle et al., 2019), or hedgehogs (Gregory and English, 1975) and infect human contacts (Gregory and English, 1975; Ansari et al., 2021).

*Trichophyton vanbreuseghemii* has a very wide gamut of hosts, including pets, farm, laboratory, and wild animals (Mariat et al., 1976; McAleer, 1980; Mitra et al., 1998). Dogs, cats (Monod et al., 2014), and horses (Chollet et al., 2015) have been implied in human contagion. Feral, hunting cats were found to be more frequently infected than domestic cats, which points to the possibility that *T. vanbreuseghemii* is contracted from rodents or soil (Drouot et al., 2009).

*Trichophyton erinacei* is the most common causative agent of ringworm in wild hedgehogs (Molina-López et al., 2012). In a French Wildlife rehabilitation center, *T. erinacei*, *T. mentagrophytes*, and *N. gypsea* were isolated from European hedgehogs. More than one-third of the animals were asymptomatic *T. erinacei* carriers, posing a latent risk of human infection (Schauder et al., 2007; Wiegand et al., 2019; Le Barzic et al., 2021). Molina-López et al. (2012), however, found neither dermatophytoses nor dermatophytes among the fungi isolated from European hedgehogs in Spain.

Several cases of animals in captivity infected with dermatophytes have been reported. Among these were siamang monkeys with cutaneous lesions from which *M. canis* was isolated (Avni-Magen et al., 2008) and a Snow Leopard with *T. mentagrophytes* that caused several human infections (Grob et al., 2018).

*Trichophyton simii* was once considered endemic in Indian subcontinent, where it was isolated from human cases (Kamalam and Thambiah, 1984), from dogs (Ranganathan et al., 1997–1998), ruminants (Mitra et al., 1998), poultry (Gugnani and Randhawa, 1973), and soil (Deshmukh, 2002). Kamalam and Thambiah (1984) documented two occurrences of families being infected by the fungus and emphasized the quick spread of the dermatophytosis between the members. *T. simii* was isolated from two hens and a pup owned by one of the families. The fungus’ endemicity is, however, in doubt since it has been reported from several countries worldwide, including Brazil, the United States, France, Belgium, Iran, and Saudi Arabia (Beguin et al., 2013). Outside the Indian subcontinent, Chabasse (1988) found the fungus in the soil in France and it was isolated from monkeys in Argentina (Boehringer et al., 1998) and Japan (Yamaguchi, 2019).

## Concluding Comments

This review article elaborates the complex relationships of dermatophyte infections with several epidemiological parameters. The latter include prevalence of the different dermatophyte species in different geographic regions and different climatic conditions, associations of age and gender with specific dermatophytes and specific clinical entities, or the frequency of exposure to contaminated environment and interaction with animals. Associations with lifestyle and effects of migration or socio-economic status are discussed, as well.

Taken all together, the review increases the relevance of dermatophyte infections in human health and well-being and suggests the need of continuing follow-up of the changes in epidemiological aspects of this group of fungi.

## Author Contributions

Both authors listed have made a substantial, direct and intellectual contribution to the work and approved it for publication.

## Conflict of Interest

The authors declare that the research was conducted in the absence of any commercial or financial relationships that could be construed as a potential conflict of interest.

## Publisher’s Note

All claims expressed in this article are solely those of the authors and do not necessarily represent those of their affiliated organizations, or those of the publisher, the editors and the reviewers. Any product that may be evaluated in this article, or claim that may be made by its manufacturer, is not guaranteed or endorsed by the publisher.
